# User emotion recognition and indoor space interaction design: a CNN model optimized by multimodal weighted networks

**DOI:** 10.7717/peerj-cs.2450

**Published:** 2024-10-31

**Authors:** Lingyu Zhang

**Affiliations:** Space Lifestyle Design, Kookmin University, Seoul, Republic of South Korea

**Keywords:** Visual emotion recognition, Self-attention mechanism, Convolutional attention module, Multimodal weighting network, Interior design

## Abstract

In interior interaction design, achieving intelligent user-interior interaction is contingent upon understanding the user’s emotional responses. Precise identification of the user’s visual emotions holds paramount importance. Current visual emotion recognition methods rely solely on singular features, predominantly facial expressions, resulting in inadequate coverage of visual characteristics and low recognition rates. This study introduces a deep learning-based multimodal weighting network model to address this challenge. The model initiates with a convolutional attention module, employing a self-attention mechanism within a convolutional neural network (CNN). As a result, the multimodal weighting network model is integrated to optimize weights during training. Finally, a weight network classifier is derived from these optimized weights to facilitate visual emotion recognition. Experimental outcomes reveal a 77.057% correctness rate and a 74.75% accuracy rate in visual emotion recognition. Comparative analysis against existing models demonstrates the superiority of the multimodal weight network model, showcasing its potential to enhance human-centric and intelligent indoor interaction design.

## Introduction

As society and the economy evolve, the convergence between interaction design and people’s daily lives intensifies, encompassing art, technology, fashion, and other influential elements. Traditional interior space design primarily caters to the functional needs of living and habitation. However, the evolution of personal aesthetics and increasing design expectations go beyond the basic level of traditional design services. Consequently, a form of interior space design that encapsulates the essence and values of its inhabitants emerges as the prospective norm in spatial design. Interaction design, as a comprehensive discipline, focuses primarily on enhancing the user experience. Presently, interaction design within interior spaces seamlessly merges design with technology. This integration leverages cutting-edge technologies like artificial intelligence, sensors, and interactive projection, among others, facilitating the fusion of users with the interior environment. The objective is to offer interior space designs that are more accessible, interactive, and tailored to individual preferences.

In contemporary interior space interaction design, visual recognition serves as a crucial tool, helping users understand and engage with interactive devices. Visual feedback mechanisms enable users to ascertain whether the system accurately interprets their body language and gestures, thereby enhancing operational precision and efficiency. Moreover, visual recognition technology allows diverse inputs to undergo analysis and recognition *via* tailored algorithms. Consequently, smart home systems can adapt and learn by capturing user behavior through cameras, furnishing users with personalized and intelligent services. Consequently, researchers and scholars have predominantly focused on enhancing the effectiveness of interior space interaction design by exploring it through the lens of user emotion recognition.

While interaction design and visual communication are integral to product interface design, their approaches differ. Interaction designers focus primarily on users’ operational habits and psychological needs, enhancing user experiences through strategic layout and interaction design. In contrast, visual communication seeks to increase the interface’s appeal and recognizability by using colors, icons, buttons, and other visual elements. Users predominantly express emotions through multiple languages, facial expressions, and body language, so relying solely on a singular perspective for emotion recognition appears limited (Chaves & Gerosa, 2021).

Research scholars have recently introduced a novel approach named the multimodal weight network (Zhou et al., 2020). This network structure encompasses diverse nodes and edges, wherein connections between nodes carry specific weights. This technique is frequently used to articulate and model complex systems that contain multiple modes or channels of information interaction. In a multimodal weighted network, nodes represent distinct entities—such as individuals, objects, or concepts—while edges signify interactions or associations between these entities. Each edge possesses a weight signifying connection strength, similarity, trust, or other interaction metrics between different entities. In multimodal weighted networks, user visual emotion recognition is a specialized node classification task that utilizes deep learning techniques to train an emotion classifier. This classifier adeptly extracts emotional cues from various facets like mouth shape, facial expressions, body language, and more, facilitating the prediction of a user's emotional state. This predictive capacity, in turn, contributes significantly to enhancing user experiences within interior interaction design.

Indeed, the use of multimodal weighted networks is instrumental in understanding and identifying users’ visual emotions. This comprehensive understanding allows for a nuanced interpretation of users’ emotional states and preferences, facilitating personalized and efficient services. Consequently, this technique introduces novel research avenues for interior space interaction design (Wiegand et al., 2019; Detjen et al., 2021).

This article proposes a deep learning-based multimodal weighted network model to address the common issue of inadequate coverage of visual features—such as facial expression information—by a single feature, which often results in suboptimal visual emotion recognition rates. This model integrates multi-weight networks and user visual emotion recognition into indoor space interaction design, culminating in an efficient and dependable interaction design framework. The key contributions of this article can be delineated as follows:

- Introduction of the self-attention mechanism: Given the model’s requirement to handle extensive input data, incorporating the attention mechanism within the visual emotion recognition model enhances the model’s comprehension of data. This augmentation significantly bolsters the model’s performance.
- Constructing visual emotion recognition using convolutional self-attention modules: By integrating a novel convolutional attention module into the visual recognition task, weight matrices are adaptively computed based on spatial pixel point relationships. Leveraging contextual relationships across different channels of the input feature map enhances the model’s ability to generate dynamic attention matrices, thereby improving the performance of user emotion recognition.
- Development of a weight network classifier that relies on optimal weights for accurate visual emotion recognition: This article analyzes original visual signals from different perspectives, assigning weights to three extracted visual signals—mouth shape, facial expression, and body language.

This article presents related work on visual emotion recognition in “Related Works”. “Methods” outlines the multimodal weighted network developed in this article, along with the user emotion recognition model. “Experiments and Analysis” describes the experimental results and discusses the scheme’s performance. “Conclusion” concludes the discussion.

## Related works

In indoor interaction design, intelligent devices play a crucial role in gathering and analyzing user visual data, utilizing recognized emotions for enhancing indoor interactions. The exploration of visual recognition has a historical trajectory spanning several decades (Yuan et al., 2022). The initial exploration of visual recognition primarily focused on expression recognition research. This involved comprehensively exploring the six common expressions in daily life and elucidating the intricate relationship between facial muscle changes and corresponding emotional states. This seminal work underscored the distinct internal human activities denoted by different expressions (Ge et al., 2022). Notably, this groundwork significantly propelled the advancement of facial emotion recognition, garnering widespread attention from researchers and laying a fundamental basis for subsequent facial expression recognition research. Traditionally, researchers heavily relied on handcrafted features like SIFT, HOG, LBP, Haar, *etc*., Adouani, Henia & Lachiri (2019), Althnian et al. (2020), Neggaz & Fizazi (2022) for processing. These features were used to train specific facial image datasets or video databases in order to develop corresponding classifiers.

In recent years, the rapid rise in the prominence of deep learning technology has attracted a wide range of research enthusiasts (Chen et al., 2018). Specifically in visual emotion recognition, neural network algorithms have acted as a crucial catalyst for advancing this field, surpassing traditional machine learning methods (Zhang et al., 2020). The proliferation and in-depth integration of deep learning across significant domains like image processing, recognition, speech recognition, and natural language processing have yielded remarkable accomplishments. The application of deep learning in visual emotion recognition primarily emphasizes two fundamental approaches: Manual extraction of image features and deep networks to discern category-specific characteristics from these features or to amalgamate diverse features. Subsequently, this amalgamation facilitates the construction of models for predicting emotional categories within speech signals. Secondly, deep networks autonomously learn and extract features about distinct emotion categories directly from original image signals. These learned features subsequently inform the development of models for predicting emotional categories.

In the realm of deep learning, convolutional neural networks (CNNs) (Zhang, Zhang & Wang, 2023) have emerged as a formidable tool for image processing. Their strength lies in the robust feature extraction capabilities embedded within their convolutional kernels, which have found widespread applications in tasks ranging from intricate image processing, pattern recognition, to feature extraction. The introduction of the FER2013 dataset, a vast collection of facial expression images initially presented by ICML, has gradually solidified its position as a key benchmark for assessing the performance of emotion recognition models (Wang et al., 2021).

As a result, there has been a significant shift in research focus, with many efforts now turning to CNNs for facial expression recognition tasks. This approach has resulted in notable advancements in performance, with single-model classification accuracies on the FER2013 dataset generally ranging from 65% to 72.7%, closely aligning with the average human performance of 65%. However, a noteworthy study (Hekler et al., 2019) takes a unique approach, leveraging three independent CNNs and their fusion to enhance performance, achieving the current best single-network accuracy of 62.44%. This analysis demonstrates the potential of ensemble methods and the integration of multiple CNNs to achieve superior results in emotion recognition tasks.

Following this, Hong et al. (2020) introduced attention convolutional networks within an end-to-end deep learning framework. Meanwhile, in Hosny, Kassem & Foaud (2020), support vector machines (SVMs) replaced the softmax layer in a deep neural network, yielding a notable classification accuracy of 71.2%. Furthermore, Bacea & Oniga (2023) innovatively proposed the Amend Representation Module (ARM) to supplant the pooling layer, resulting in elevated test accuracy.

Another study (Khaireddin & Chen, 2021) delved into comparing the performance of three distinct architectures: VGG (Zhou et al., 2020), Inception (Si et al., 2022), and ResNet (Yu et al., 2023). The findings indicated VGG achieving the highest accuracy at 72.7%, closely trailed by ResNet at 72.4%, with Inception securing 71.6%. In the evolution of expression recognition, some researchers underscore the limitations of relying solely on a static picture for expression recognition, overlooking inherent dynamic patterns within facial behaviors (Yu et al., 2023). To address this concern, Norouzi et al. (2023) conceptualizes facial expression as a sequence of actions occurring within consecutive or overlapping temporal intervals of facial movement. They proposed the Interval Time-constrained Boltzmann Machine (IT-RBM), which adeptly captures spatial and temporal information of facial behaviors for facial expression analysis, culminating in remarkable performance outcomes.

In the interactive design of interior space, we need to collect a large amount of user data, including user actions, voice, facial expressions and so on. However, collecting this data directly can create a huge volume of data that is difficult to process. At this time, Jiang et al. (2024) uses compressed sensing technology to recover complete user data from fewer measured values by utilizing the sparsity of data, so as to achieve data compression and efficient processing. In Sun (2024), through image reconstruction technology, the indoor environment can be displayed in the form of three-dimensional images, so that users can more intuitively understand the layout and decoration of indoor space. Indeed, while the current landscape of expression recognition research has flourished, propelled by the integration of deep learning methodologies, significant challenges persist in attaining a practical, highly accurate, and robust expression recognition model. These challenges stem from substantial intraclass differences and subtle interclass distinctions observed within facial expressions.

Recent efforts have involved the integration of multimodal weighted networks across various domains. Liu et al. (2021) notably introduced these networks into speech emotion recognition, developing deep learning-based models. Meanwhile, Wang et al. (2022) expanded on this by leveraging multimodal weighting networks for web-based speech’s multimodal emotion recognition, encompassing speech, intonation, and linguistic elements. Although these approaches pave a path for visual emotion recognition, their application remains confined to experimental research stages, posing challenges for real-life deployment. Furthermore, there is considerable room for improvement in multimodal consideration, particularly in incorporating aspects like a user’s body language, mouth shape, facial expressions, *etc*., within visual recognition. This area merits deeper research and exploration for enhancing the effectiveness and applicability of visual emotion recognition models.

## Methods

**Figure 1:**
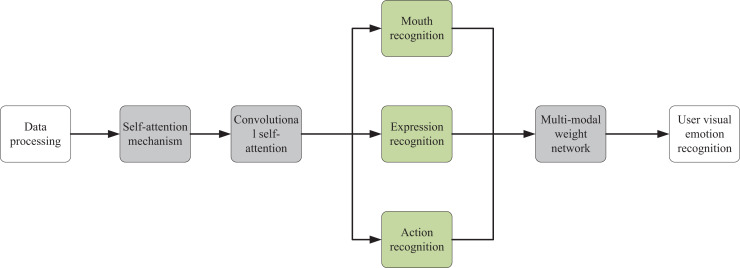
Model frame drawing.

The indoor interaction system presented in this article is primarily designed around users’ visual emotion recognition. In emotional recognition tasks, models that utilize non-local feature fusion networks can build convolutional attention modules based on the self-attention mechanism to identify users’ emotional expressions. This type of model can effectively capture the interactions between different regions of the image and extract key features related to emotions. These features can then be input into a multimodal weighted network to generate the final distribution of user emotions. In contrast, traditional CS models may focus more on data storage and retrieval rather than feature extraction and fusion in emotional recognition tasks. Therefore, as illustrated in Fig. 1, after image data collection, a visual emotional recognition model based on convolutional attention modules is primarily constructed on the foundation of the self-attention mechanism. This model can recognize three emotional expressions of users and finally input the extracted visual signals into a multimodal weight network to obtain the final distribution of user emotions.

### Self-attention mechanism

**Figure 2:**
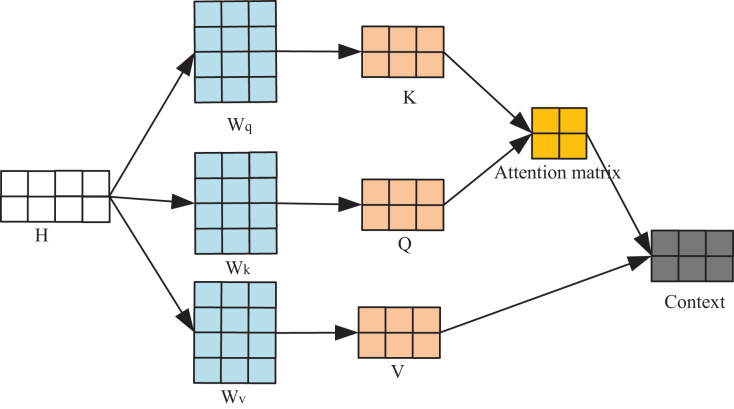
Flow chart of self-attention mechanism.

This section introduces the self-attention mechanism to enable the model to prioritize and retain crucial information within the training data. This mechanism operates by computing Query-Key-Value (QKV) associations, allowing the model to enhance its data comprehension by identifying and focusing on key elements within the input. As shown in Fig. 2, the input $H = [{h_1},{h_2}]$, each row therein represents a corresponding input vector. The three-parameter matrix ${W_q},{W_k},{W_v}$ in the figure serves to transform the input H into the corresponding query spaces $Q = [{q_1},{q_2}]$, $K = [{k_1},{k_2}]$ and $V = [{v_1},{v_2}]$, and the transformation process is as follows:

(1) $$[{q_1} = {h_1}{W_q},{q_2} = {h_2}{W_q}] \to Q = H{W_q}$$

(2) $$[{k_1} = {h_1}{W_k},{k_2} = {h_2}{W_k}] \to K = H{W_k}$$

(3) $$[{v_1} = {h_1}{W_v},{v_2} = {h_2}{W_v}] \to V = H{W_v}$$

The computation of the output context, following the application of the attention mechanism, relies on the three feature matrices: *Q*, *K*, and *V*. This resultant output encapsulates the model’s understanding of the input *H*, constructed upon the foundation of the self-attention mechanism. Each line of this output $contex{t_i}$ signifies the model’s focal points at corresponding positions ${h_i}$ within the input sequence. The process of computing the context unfolds as follows:

(4) $$context = soft\max {\left(\displaystyle{{Q{K^T}} \over {\sqrt {{D_k}} }}\right)^V}$$

where ${D_k}$ is determined by the dimensionality of the vector *Q*.

### Visual emotion recognition based on convolutional attention module

To address the inefficiency of the feature extraction process caused by the computational invariance of convolution, which results from its invariant parameters. We introduce a new convolutional attention module (AM) for the visual recognition task. According to user emotion recognition performance requirements in indoor spatial interaction, we detect the user’s mouth shape, facial expression, and body movement in three directions.

Given the inherent specificity of the visual recognition task, the expressions of a human face are often closely related to our five senses, particularly the eyes and mouth. The expressions of body movements are also associated with our hands, legs, neck, *etc*. To capture the specific details of the user’s face and limbs, we analyze the relationship between pixel points in the input image and adaptively compute the weight matrix based on the spatial relationships between these pixel points. The rich contextual relationships embedded between different channels of the input feature map are also considered to guide learning the dynamic attention matrix. By utilizing two sets of 1 * 1 convolutions and updating the parameters of the convolution kernel corresponding to each convolution window, the attention is computed while extracting the global features of the input feature map. Eventually, during the aggregation process, different weights are assigned to each pixel point of the input feature map, *i.e*., different attention is assigned to different input locations. This structure allows the module to be efficiently utilized in traditional convolutional architectures while being more efficient in feature extraction.

**Figure 3:**
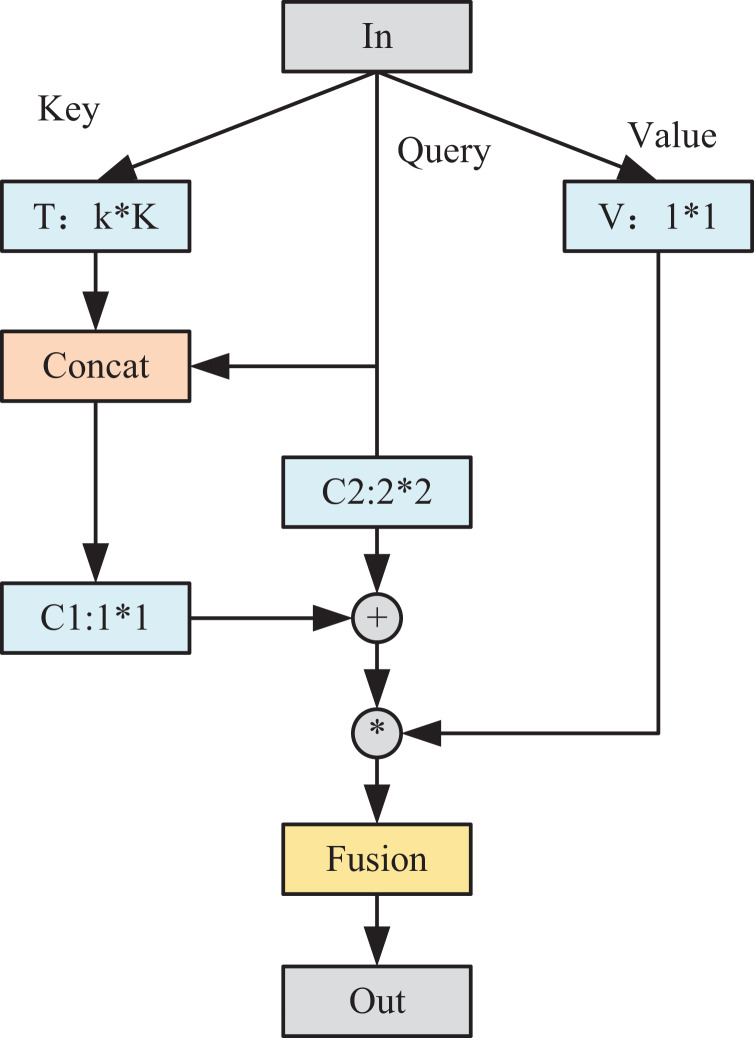
Structure diagram of AM module.

In Fig. 3, the AM module unifies the exploration of contextual information and the acquisition of the self-attention mechanism within a cohesive architecture. This integration maximizes the use of abundant contextual details among adjacent keys, effectively enhancing the learning process of self-attention. Consequently, this amalgamation enhances the module’s capacity to characterize and discern nuanced features within the output. Set the input feature map $In$ as $H \times W \times C$. First, we spatially convolve each key using the $k*k$ convolution set, and denote the learned context information as T, which reflects the static context information between different channels. The attention mechanism comprises two distinct components, both involving independent convolution operations. Initially, the acquired static context information T is concatenated with the query to enrich the characterization of information among contexts. Subsequently, employing a convolutional group facilitates the learning process of attention, resulting in the derivation of the attention matrix A1.

(5) $$A1 = [T,Q] \otimes C1.$$

Meanwhile, to reinforce the input information to some extent, we use another independent $1*1$ convolutional group for query using the same way of learning attention to get the attention matrix A2:

(6) $$A2 = Q \otimes C2.$$

The two sets of attention matrices were subsequently fused to obtain the final attention matrix A3.

(7) $$A3 = A1 + A2.$$

The attention-based output T1 is obtained by aggregating A3 with V:

(8) $$T1 = V \times A3.$$

Ultimately, the static information T undergoes fusion with the contextual representation T1, leveraging the dynamic attention mechanism, culminating in the final output of the module. Subsequently, a visual recognition network model is devised, incorporating the ATB module and its resultant output. This network encompasses four distinct sets of convolutions along with one fully connected layer. Each convolutional set comprises varying numbers of layers—specifically, 2, 2, 3, and 3 convolutional layers, respectively.

### Multimodal weighted network coding classification model

After feature extraction from visual emotion recognition based on convolutional attention modules, the multi-modal weight network encoding classification model aims to deeply analyze the originally captured visual signals from various dimensions. This model comprehensively considers multiple features and automatically assesses the importance of each individual feature through the network, leading to more accurate and comprehensive emotional classification results.

**Figure 4:**
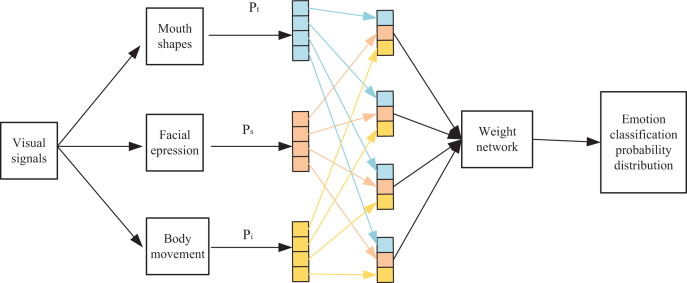
Multi-modal weight network classification model prediction process.

Illustrated in Fig. 4, the model initially transforms user mouth shape information within the visual signal into textual information through mouth shape recognition. Subsequently, this process facilitates the derivation of the probability distribution for emotion classification:

(9) $${P_t} = ({t_1},{t_2},{t_3},{t_4}).$$

The user expression information f in the visual signal is used as an input to the overall model, and AM obtains a probability distribution for visual emotion classification:

(10) $${P_s} = ({s_1},{s_2},{s_3},{s_4}).$$

A probability distribution is also obtained based on the user’s body movement information in the visual signal:

(11) $${P_i} = ({i_1},{i_2},{i_3},{i_4}).$$

The prediction generated by each individual model represents the expression of a distinct feature in sentiment categorization. Dynamically assigning varying weights to the predicted outcomes of these different models entails dynamically altering the influence of each single feature within the overall categorization process. This dynamic adjustment aims to further refine the accuracy of sentiment categorization.

In this context, the probability distributions of each component from the individual models are utilized within the weighting network. These components play a role in linearly regressing the respective elements of the final classification probability, as described by Eq. (12).

(12) $${\varphi _n} = {\alpha _{n1}}{s_n} + {\alpha _{n2}}{t_n} + {\alpha _{n3}}{i_n} + {g_n}$$

where *n* represents the emotion category. This article is a four-category situation (emotion categories are angry, happy, sad, and neutral), so $n = \{ 1,2,3,4\}$. ${s_n},{t_n},{i_n}$ denote the n-th category probability in ${P_s},{P_t},{P_i}$ respectively. ${\alpha _{n1}},{\alpha _{n2}},{\alpha _{n3}}$ and ${g_n}$ are the learnable weights and bias terms that balance the three. For example, ${s_1}$ denotes the probability that the emotion category obtained from the visual signal after AEM is angry in this article. ${\varphi _1}$ denotes the probability that the category of anger is obtained after automatic weighting regression by the weighting network. Since it is a four-category case, the categorization probability $P = ({\varphi _1},{\varphi _2},{\varphi _3},{\varphi _4})$ is obtained. After softmax normalization finally get the probability distribution of emotion classification of speech signal $\tilde P = ({\tilde \varphi _1},{\tilde \varphi _2},{\tilde \varphi _3},{\tilde \varphi _4})$.

## Experiments and analysis

In this section, we engage in simulation experiments to evaluate the performance of the user visual emotion recognition model under the multimodal weighting network. We assess its efficacy alongside prevalent network models such as convolutional neural network (CNN) (Zhang, Zhang & Wang, 2023), GoogleNet (Jiang et al., 2024), VGG+SVM (visual geometry group+support vector machine) (Sun, 2024), CNN+SVM (Yang et al., 2023), and ResNet (Yu et al., 2023). Our objective is to compare and analyze the experimental outcomes of each method type. Ultimately, we present the confusion matrix alongside the correctness curve graph derived from this article’s scheme (Donuk et al., 2023).

These experimental results serve a dual purpose: first, to analyze the impact of the multimodal weighted network and convolutional attention module on user emotion recognition performance; and second, to investigate how the introduction of the multimodal weighted network and user visual emotion recognition, based on the convolutional attention module, influences indoor space interaction design (Lyu et al., 2023).

### Evaluation indicators

The prediction evaluation metrics used in this article are accuracy (ACC), precision (Pre) and recall (R). Let true positive (TP) denote that positive samples are predicted as positive samples, false negative (FN) denote that positive samples are predicted as negative samples, false positive (FP) denotes that negative samples are predicted as positive samples, and true negative (TN) denote that negative samples are predicted as negative samples. Then:

(1) The correct rate metric indicates the proportion of positive and negative samples correctly categorized in the user’s visual emotion categorization recognition. The formula is as follows:

(13) $${\rm ACC = }\displaystyle{{{\rm TP + TN}} \over {{\rm TP + TN + FP + FN}}}.$$

(2) The accuracy rate metric is for the user visual emotion classification recognition results and indicates the proportion of samples predicted to be positive that are positive. The formula is as follows:

(14) $${\rm Pre} = \displaystyle{{{\rm TP}} \over {{\rm TP + FP}}}.$$

(3) The recall metric is for the visual emotion raw labels and indicates how many positive classes in the visual signal sample were predicted correctly. The formula is calculated as follows:

(15) $${\rm R} = \displaystyle{{{\rm TP}} \over {{\rm TP + FN}}}.$$

### Parameterization

This study involves experimentation conducted on the HMDB (https://zenodo.org/records/5758926, doi: 10.5281/zenodo.11046710) and CK+ (https://zenodo.org/records/11221351, doi: 10.5281/zenodo.11221351) datasets. The initial dataset is stored in. H5 format before commencing the experiments. The experimental setup utilizes a 2020Ti graphics card running the PyTorch framework. The training employs an SGD iterator over 300 rounds, incorporating a 0.9 momentum factor, 0.0005 weight decay, and a learning rate 0.1. During the training process, an adaptive adjustment of the model occurs every ten rounds after the initial 80 rounds, contingent upon the change in loss observed.

In the network architecture constructed, traditional convolution is exclusively employed solely within the initial layer of the first convolution set. All subsequent convolution operations are executed utilizing the AM module. Adopting traditional convolution in the initial layer augments the number of channels in the input feature maps, thereby facilitating model construction and subsequent processing *via* the AM module. The ultimate fully connected layer is tailored based on the fixed count of specific expression categories set at four.

In terms of data processing, this article loads raw data from HMDB and CK+ datasets. For video dataset HMDB, key frames or video fragments are extracted. For the image dataset CK+, the image file is loaded directly. At the same time, the pixel value of the image is normalized to the range of [0, 1] or [−1, 1] to eliminate the difference in brightness, contrast and other aspects of different images.

### Comparative experimental analysis

**Figure 5:**
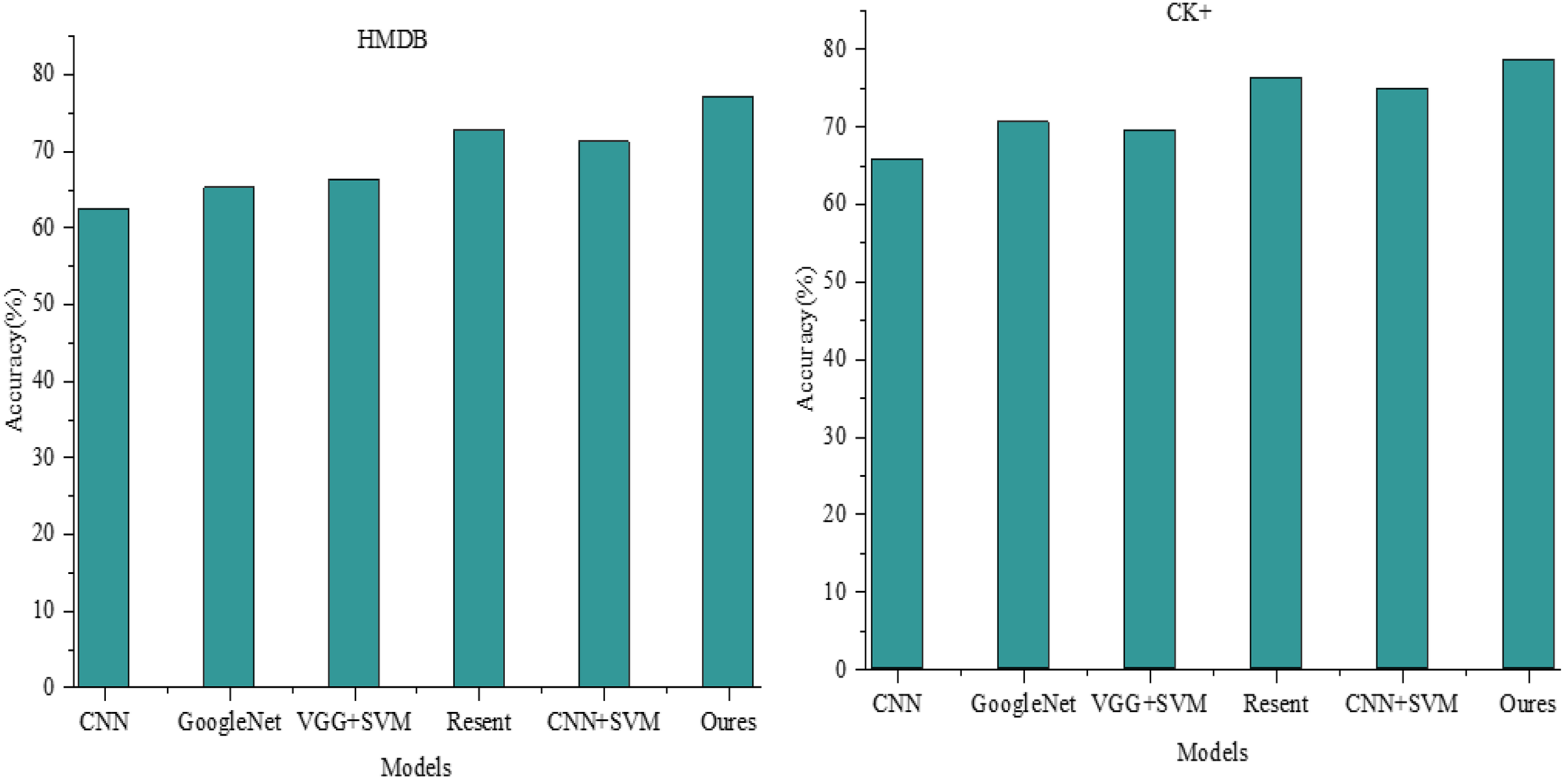
ACC comparison among different models.

The final recognition accuracy achieved by our model stands at 77.057% and 78.562% on the test sets of the HMDB and CK+ datasets, respectively. In our comparative analysis against current leading methodologies, presented in Fig. 5, our proposed approach demonstrates substantial advancements in expression recognition tasks relative to traditional and contemporary deep learning methods.

Specifically, on the HMDB dataset, the CNN model achieves a correctness rate of 62.44%. Comparatively, GoogleNet, VGG+SVM, and CNN+SVM outperform the CNN model, achieving correctness rates of 65.20%, 66.31%, and 71.20%, respectively. ResNet exhibits a correctness rate of approximately 72.7%, fairly comparable to our proposed model’s prediction. However, with an expansion in the test dataset, a declining trend is noticeable in the accuracy of ResNet due to the absence of a multimodal weight network, resulting in its incapability to accurately recognize user emotions amid increased variation in user features like limbs, mouths, and facial expressions within the dataset.

Conversely, our model’s performance advantage is less pronounced within the CK+ dataset, primarily comprising facial expression images. The accuracy of ResNet is particularly noteworthy, reaching 76.251%, exhibiting a marginal difference of approximately 2.3% from our model’s performance.

**Figure 6:**
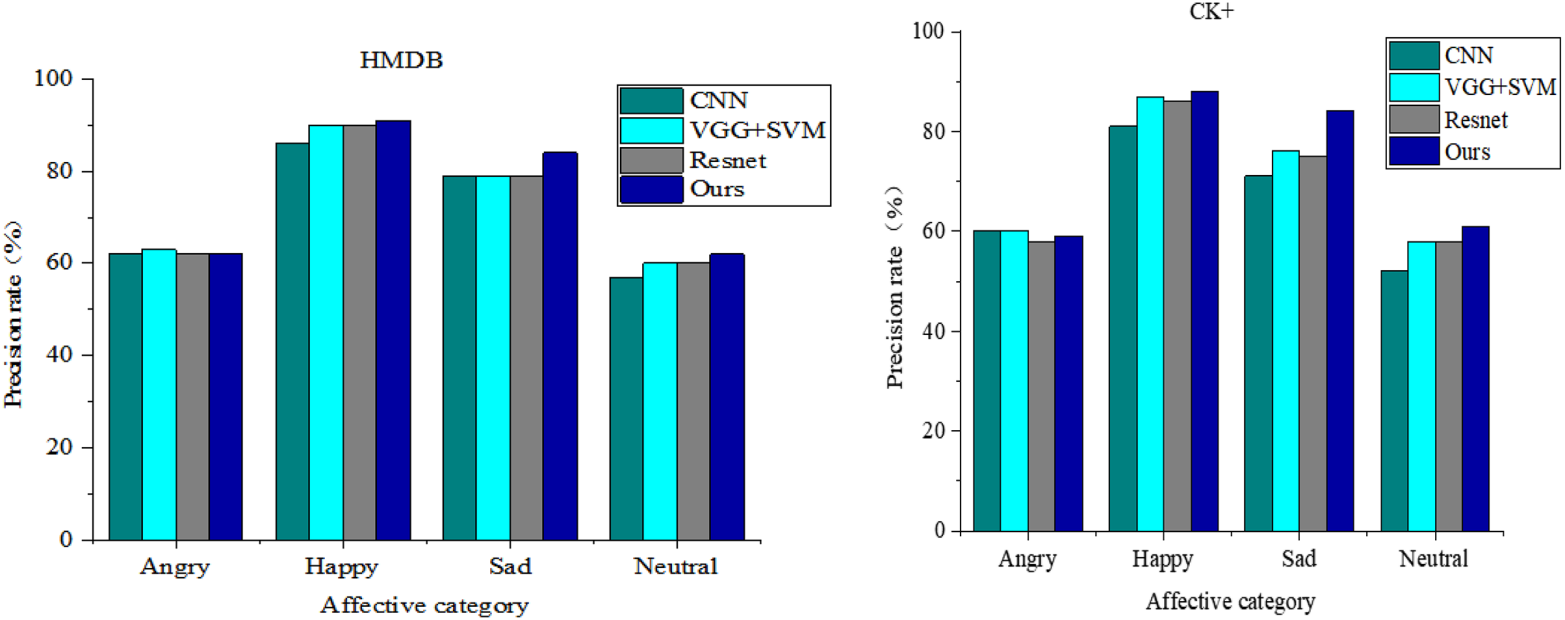
Comparison of sentiment recognition performance under different models.

To comprehensively illustrate the expression recognition outcomes in this study, we present a longitudinal comparison across different models concerning the HMDB and CK+ datasets. The dataset undergoes classification, wherein the models’ classification accuracy for each expression category is compared. Figure 6 showcases the recognition accuracy rates of each model for the four visual emotion categories alongside their final average accuracy rates. Given the distinct experimental environments and hyper-parameter configurations, the comparison schemes for visual emotion recognition experiments include the CNN network, VGG+SVM, and ResNet. Notably, VGG+SVM achieves a recognition accuracy of 73%. However, the model proposed in this article attains the highest average accuracy of 74.75% across both datasets.

Regarding specific expression categories, the network models introduced in this article exhibit superior recognition rates for all expressions except for the anger and fear categories, where the recognition performance slightly trails that of the VGG+SVM network. Notably, our approach achieves a 91% recognition accuracy for the joy class of emotions. The substantial improvement observed in this article’s model for the sadness class of emotions is particularly noteworthy, showcasing a 5% enhancement over the best-performing ResNet.

**Figure 7:**
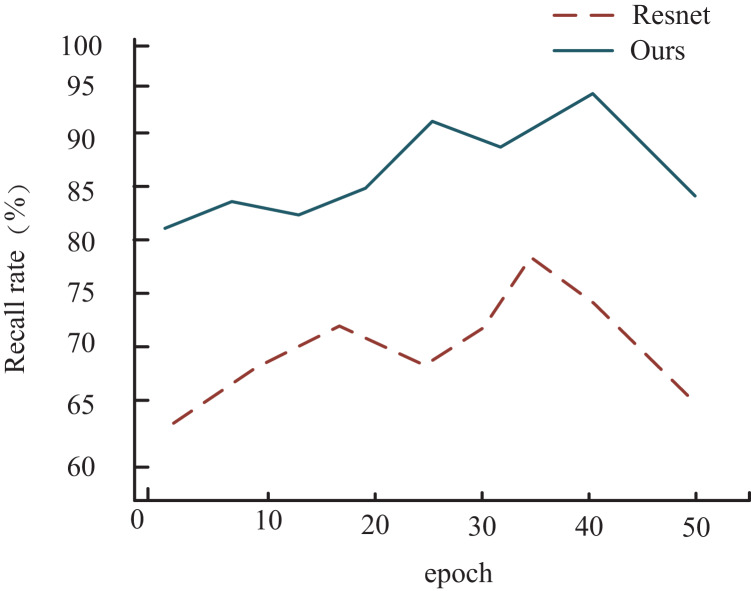
Comparison of recall rate among different models.

To provide a clearer visual representation of accuracy progression during training, we present a graph that illustrates the changes in recall on the HMDB dataset for both the proposed method in this article and the ResNet network model, which shows a similar performance trajectory. As depicted in Fig. 7, it’s evident that for both models, the recall undergoes a rapid increase during the initial training phase, followed by a gradual convergence and eventual decline. However, a notable distinction arises from incorporating a multimodal weighting network in our proposed model.

In this context, the recall of the model outlined in this article showcases a delayed onset of decline compared to ResNet during training. Overall, the model presented in this article exhibits significantly superior performance compared to ResNet, mainly attributed to integrating the multimodal weighting network.

### Ablation experiments

**Figure 8:**
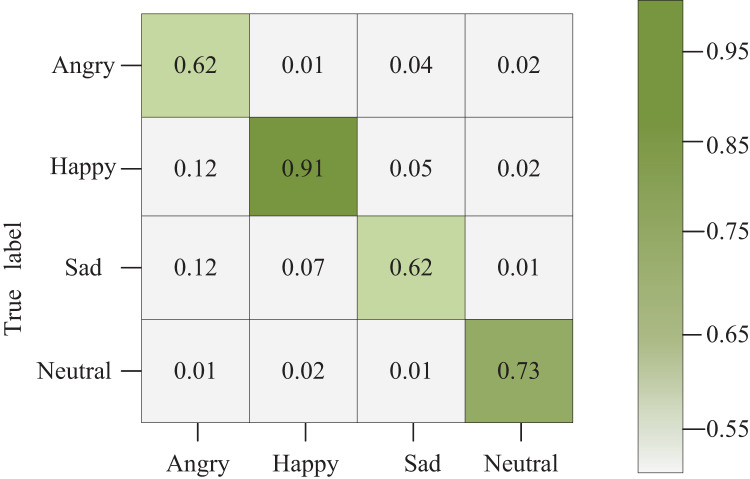
Confusion matrix graph.

Figure 8 illustrates the confusion matrix corresponding to the network model constructed in this article. Diagonal squares denote the accurate recognition of the current emotion. In contrast, squares off the diagonal represent the probability of recognizing the emotion on the vertical axis as the emotion on the horizontal axis. The shading in the grid varies according to the values within each grid.

Notably, our model achieves a 62% recognition rate for emotions categorized as sadness compared to other expressions. This outcome stems from the significant similarity observed in daily life among fear, sadness, and anger. These expressions often share notable similarities in body and facial features, making it challenging even for neural networks to extract distinct features accurately, especially when data is limited. This similarity complicates accurate feature extraction by the neural network, contributing to the observed recognition rate in the sadness category.

**Figure 9:**
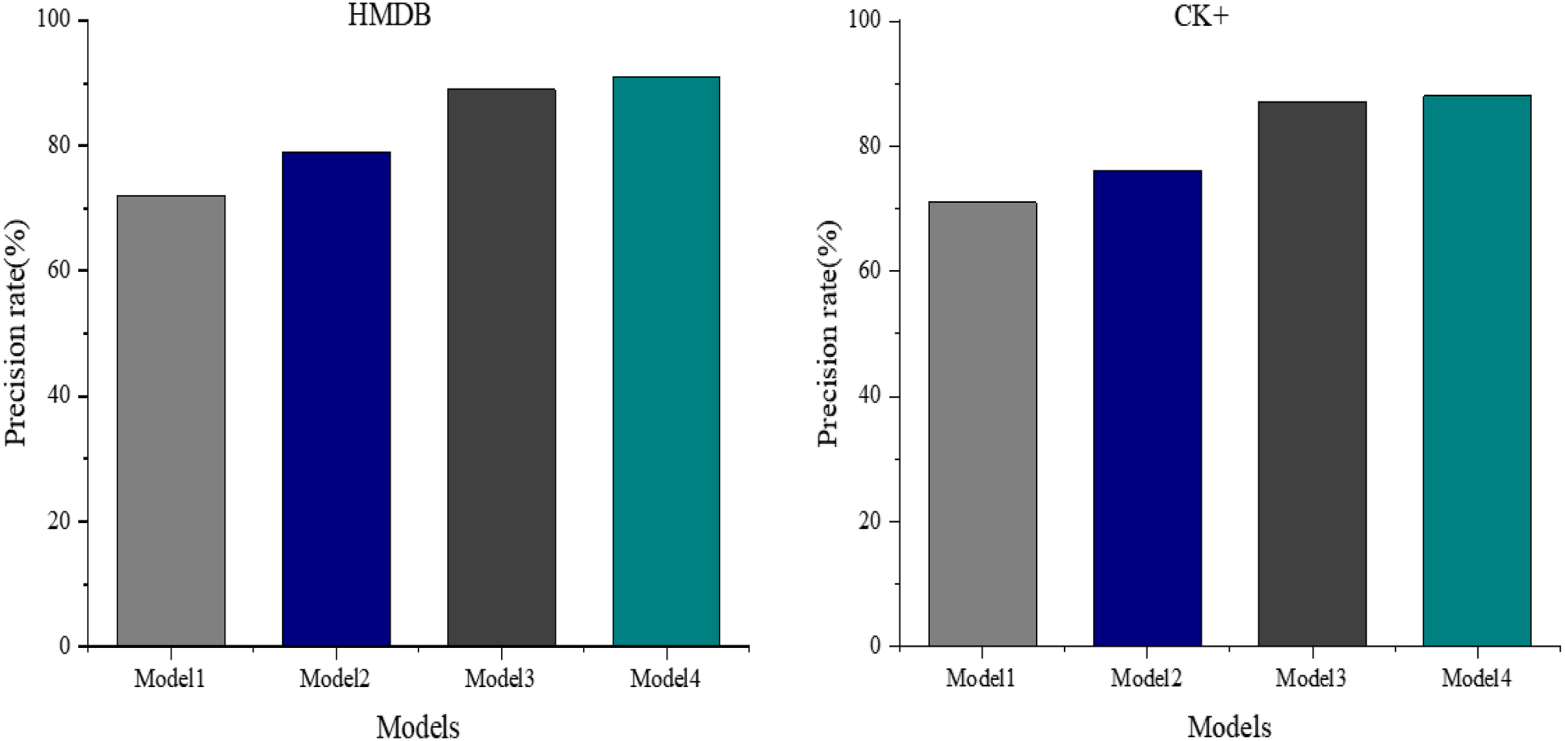
Comparison of model precision under different modules.

Figure 9 displays the ablation experiments conducted on the glad dataset to assess and evaluate the neural network model’s performance. These experiments aimed to understand the impact of the self-attention mechanism and the multimodal weight network coding classification on the model’s efficacy by progressively removing them.

The results depict a discernible effect on recognition accuracy when removing these components. Model 3, derived from Model 4 by removing the self-attention mechanism, attains a recognition accuracy of 89%. Conversely, Model 2, obtained by exclusively eliminating the multimodal weight network coding classification from Model 4, experiences a substantial drop in recognition accuracy, achieving only 79%.

Furthermore, when both the self-attention mechanism and the multimodal weight network coding classification are removed from the proposed Model 4, resulting in Model 1, the recognition accuracy plunges to a minimum of 72%. These findings underscore the significant contributions of both the self-attention mechanism and the multimodal weight network coding classification to the model’s overall performance in visual emotion recognition.

### Discussion

“Comparative experimental analysis” and “Ablation experiments” present a comprehensive analysis of the comparative performance of the proposed schemes outlined in this article. Examining three crucial performance metrics—correctness, precision, and recall—reveals the substantial superiority of the scheme introduced in this article, achieved through incorporating a multimodal weight network. This scheme attains a correctness rate of 77.057% and a precision rate of 74.75% on the dataset. Notably, the recall rate peaks at 94.7% during the training process, showcasing a significantly enhanced performance compared to CNN, VGG+SVM, ResNet, and other models.

The confusion matrix diagram for the network model constructed in this article further supports its accuracy in classifying gladness, achieving an impressive recognition rate of 91%. This holds immense significance, particularly in indoor space interaction design. Accurately recognizing emotions, particularly happiness, in visual emotion recognition among indoor users bears substantial value. This recognition aids designers in better-comprehending user needs and emotions, facilitating the provision of more personalized and comfortable designs. By monitoring users’ visual responses, designers can discern which design elements evoke positive emotions like pleasure, excitement, or comfort and negative emotions such as dissatisfaction, despondency, or fatigue. This profound understanding of user emotions empowers designers to optimize their designs effectively, aligning them more closely with users’ emotional requirements.

Visual, emotional recognition is a pivotal tool for designers to elevate both their designs’ artistic appeal and functionality. Exceptional interior design transcends mere functionality by evoking emotional resonance within users. Through the lens of visual emotion recognition, designers gain valuable insights into which design elements effectively convey desired emotions and atmospheres. This understanding empowers them to integrate these emotions seamlessly into their designs, enhancing both their creations’ artistry and functionality.

Moreover, visual emotion recognition technology enables designers to identify which colors, shapes, materials, and layouts best evoke specific emotional responses by testing and analyzing users’ reactions to design elements. This technology makes design more targeted and precise, helping designers achieve artistic and innovative outcomes. Through visual emotion recognition, this article allows designers to gain a deeper understanding of users’ emotional needs, thereby integrating more emotional elements into their designs. This emotional connection with users can enhance their sense of identity and satisfaction with the design, ultimately strengthening brand image and loyalty. While visual emotion recognition technology has made certain progress, it still faces numerous technical challenges. For instance, accurately identifying and understanding complex emotional responses, as well as addressing emotional differences across various cultures and backgrounds, is crucial. Since emotional differences in different cultural contexts may affect the results of visual emotion recognition, future research will continue to focus on visual emotion recognition in cross-cultural contexts, further exploring the interaction between emotions and cognition, and how this interaction affects users’ experience and satisfaction, ultimately providing design services that better meet user expectations.

## Conclusion

This article delves deeply into enhancing intelligent design within indoor space interaction by extensively investigating user visual emotion classification, employing a multimodal weight network. Integrating the self-attention mechanism into the convolutional network has paved the way for developing a visual emotion recognition network based on the convolutional attention module.

Our approach harnesses the user’s facial expression, mouth shape, and body features extracted from the visual emotion recognition network as inputs into the multimodal weighting network. This network’s weighted linkage yields the final probability distribution of the user’s emotional state. Notably, the experimental outcomes reveal the superiority of our method over prevalent models like CNN, VGG+SVM, CNN+SVM, and ResNet. Achieving significant advancements in correctness, precision, and recall, our method attains a correctness rate of 77.057% and a precision rate of 74.75%, surpassing the average performance of comparative models. ResNet mainly demonstrates a 3.2% to 4.3% higher precision rate than the comparison models on average.

Our model excels in classifying and recognizing happiness during the training process, allowing designers to identify design elements that evoke positive emotions in users. Consequently, designers can adjust these elements to amplify positive emotions or alleviate negative ones, enhancing user experience and satisfaction. In the future, we will further improve the construction of visual emotion recognition module and improve the performance of emotion recognition model, so as to more accurately identify the emotional needs of users, help designers provide more intimate and comfortable design services, and enhance user experience and satisfaction.

## Supplemental Information

10.7717/peerj-cs.2450/supp-1 Supplemental Information 1 This is code.

## Additional Information and Declarations

### Data Availability

The following information was supplied regarding data availability: The code is available in the Supplemental File. The Indoor Location & Navigation data is available at Kaggle: https://www.kaggle.com/c/indoor-location-navigation/overview. The data is available at Zenodo: Hu, Y., Fan, X., Yin, Z., Qian, F., Ji, Z., Shu, Y., Han, Y., Xu, Q., Liu, J., & Bahl, P. (2023). Indoor Location Competition 2.0 Dataset [Data set]. The 29th Annual International Conference On Mobile Computing And Networking (MobiCom 2023), Madrid, Spain. Zenodo. https://doi.org/10.5281/zenodo.8265879. The HMDB XML is available at Zenodo: - Hirschey, M. (2021). HMDB Clean (21Q4) [Data set]. Zenodo. https://doi.org/10.5281/zenodo.5758926. - D, A., Ojjela, O., & Poulose, A. (2024). Enhancing Facial Emotion Recognition: A Comparative Analysis of Sobel and Laplacian Filters for Computer Vision Applications [Data set]. Zenodo. https://doi.org/10.5281/zenodo.11046710 The CK+ dataset is available at Zenodo: None. (2024). CK+ [Data set]. Zenodo. https://doi.org/10.5281/zenodo.11221351.
